# How to promote the social–emotional competence of rural left-behind children? An empirical study based on propensity score matching

**DOI:** 10.3389/fpsyg.2023.1052693

**Published:** 2023-03-03

**Authors:** Xiaolan Mo, Gaojun Shi, Yanan Zhang, Xiao Xu, Chengjun Ji

**Affiliations:** ^1^Jing Hengyi School of Education, Hangzhou Normal University, Hangzhou, China; ^2^China Educational Modernization Institute, Hangzhou Normal University, Hangzhou, China

**Keywords:** left-behind children, social–emotional competence, teaching support, propensity score matching, in rural

## Abstract

Social–emotional competence (SEC) played an important role in promoting the physical and mental development of children, but there exist huge gaps in SEC development between rural left-behind children. This study used propensity score matching (PSM) to investigate 578 rural children about the effects of being left behind as well as individual characteristics and teacher support on their development of SEC. The results showed that being left behind had significant negative effects on the SEC of rural children. The development of SEC varies among left-behind children of different genders and length of left-behind duration. Teacher support had a significant moderating effect on the influence path of SEC, which could effectively mitigate the negative effects of left-behind children. Therefore, this study played an implicative role in studying the development of left-behind children’s SEC. The government and society should provide adequate cultural capital by completing the support system for compensating the lack of cultural capital. Schools and teachers should pay more attention to the development of left-behind children’s SEC through curriculum development and performance evaluation to create a positive atmosphere. Parents should promote SEC development for left-behind children by improving their communication and family parenting styles.

## 1. Introduction

Labor migration had become a common phenomenon in the world, with parents seeking to reduce family poverty and increase their income by working outside, which may prevent some parents from bringing their children with them at the stage of compulsory education becoming left-behind children (Li et al., 2015; Hu et al., 2020). The World Children’s Fund defined left-behind children as all children who experience unequal treatment in health, education, and welfare (Antia et al., 2020). According to Fan et al. (2010), left-behind children were defined as those under the age of 16 years whose both parents had been away for work for 6 months or more, or one of whose parents stayed at home while was unable to take care of them. The number of left-behind children was quite large worldwide, with Asia being the main gathering place for left-behind children and more than 12 million left-behind children in the compulsory education age group in China. According to the survey report (On the Road to School, 2018), 96% of left-behind children in rural areas were guarded by their grandparents and 4% are under the guardianship of other relatives and friends.

It was difficult for parents to accompany and guide left-behind children timely as they were separated from them for a long time, which may result in the insufficient social and emotional development of left-behind children. According to Denham et al. and Wang et al., social–emotional competence (SEC) referred to the knowledge, attitudes, and skills that an individual acquires and effectively regulates emotions. SEC included self-awareness, self-management, social awareness, interpersonal skills, and responsible decision-making (Denham et al., 2003; Wang et al., 2017). SEC was considered a multi-level comprehensive concept, which avoided interpreting “sociality” as the ability to adapt to society and “emotion” as a personal emotional trait.

The research showed that the SEC has an important role in students’ study and life and could positively predict students’ academic achievement (Rhoades et al., 2011), attitude (Schonert-Reichl, 2019), and behavior (Yang et al., 2019). At the same time, SEC was supposed to be associated with parental involvement and parenting styles (Kwon et al., 2013), being affected by parental care and companionship to a large degree, implying that lack of parental care negatively impacted child development (Antman, 2012). However, there were few studies on children’s SEC, which had not been paid enough attention to. The difference in SEC between left-behind children and non-left-behind children, as well as the possible compensation path, needs to be further explored. This study raised the following research questions:

1. What were the impacts of left-behind experience on children’s SEC?
2. How do family factors affect children’s SEC?
3. What was the compensation path to provide positive assistance for rural left-behind children’s SEC?

To this end, this study summarized the literature reviews on the SEC of left-behind children and put forward hypotheses in the next section. According to the internationally recognized SEC questionnaire, a sample investigation was conducted among the rural left-behind children to explore the relationship between the research variables in Section 3. The propensity score matching (PSM) method was used to present the analysis results in Section 4, which eliminates the endogenous problem caused by the selection bias. The results of this study are discussed in Section 5, and the implications are put forward in Section 6.

## 2. Literature review and research hypothesis

Some previous research results showed that the SEC of rural left-behind children in China was in a weak position (Cadsby et al., 2020; Wang et al., 2020; Gu, 2022). Qian et al. (2021) used the score-matching method to analyze the tracking survey data on China’s education and found that, despite overcoming the endogenous situation of parents choosing to go out to work, the peer relationship of left-behind children was significantly weaker than that of non-left-behind children. The previous study also found that being left behind could significantly increase children’s negative emotions and negatively affect their sense of self-efficacy (Zhang et al., 2019). Cui and Xu (2021) analyzed the tracking survey data of families and found that parents’ going out had a significant impact on the non-cognitive ability of left-behind children. Specifically, the score of left-behind children in emotional stability and openness of performance was lower than that of non-left-behind children. Based on the qualitative study method of interventionism, Xiao and Liu (2022) interviewed teachers, parents, and other family members of left-behind children and found that the left-behind children lacked family cultural capital support, which affected children’s adaptability and overall performance in school. Wang et al. (2019) investigated 2,996 fourth-and fifth-grade students in western China and found that the left-behind was one of the important reasons for the backward development of the SEC. A few other studies have found that, however, left-behind children in the development of the SEC were not significant (Peng and Dai, 2019).

According to the theories of social development such as social cognitive development theory represented by Jean Piaget (Barrouillet, 2015) and social learning theory represented by Bandura (1999), family environment, parental companionship, and parent–child relationship were considered to play vital roles in children’s development, which naturally leads to the disadvantageous position of the growth of left-behind children. According to the family stress theory (Patterson and Garwick, 1994), the parents of left-behind children were often faced with high and persistent pressure such as low income, child-rearing, and career development. Family care stress and work stress significantly positively predicted violence between migrant couples with especially family care stress being more likely to evolve into marital conflict (Perry-Jenkins and Gerstel, 2020). Moreover, poor parent relationships will exert a significant negative effect on children’s social behavior and SEC (Richmond and Stocker, 2008), which made left-behind children suffer from the direct and indirect effects of family pressure. In addition, some studies suggested that the increase in family income had a positive effect on the stability of family and marriage (Arnold and Beelmann, 2019), which could make up for the negative effect of the separation of parents and children. However, comparing different groups of children aged 3–15 years, including migrant children, left-behind children, and rural and urban children in non-migrant families, Lu et al. (2019) found that the increase in *per capita* income of left-behind families could not offset the negative effects of left-behind families on children. On the contrary, the research by Tang et al. (2018) further indicated that the improvement in economic conditions would have a negative impact on left-behind children who lack certain guidance and constraints. To sum up, the development of children’s SEC needed a good family atmosphere, parent–child relationships, which had a larger functional space in the development of children (Li and Qiu, 2016). SEC was developed within the context of a responsive and sensitive parent–child relationship (Lengua et al., 2007). Therefore, compared with non-left-behind children, the SEC development of left-behind children was more likely to be the direct sufferer of family structure dismemberment and shallow emotional support.

In addition to the influence of family on the development of children’s SEC, some research emphasized the important role of the school. Jeon et al. (2019) conducted three-level multi-level analyses and found that teachers, as key participants in children’s development and an important guide in life-learning behaviors, play an increasingly important role in children’s development. A study by Soriano-Ayala and Cala (2017), on the impact of SEC on adolescents in Spain, found that schools play an important role in the development of adolescents’ SEC development. Collie (2022), using survey data from some secondary school students in Australia, further analyzed and found that positive teacher support was an important predictor of SEC and contributes to the development of children. Especially for the left-behind children, after the modulation of the teacher support, the differences between the left-behind children and the non-left-behind children in the aspects of emotion and behavior disappeared (Fan et al., 2010). Therefore, we believed that teacher support was indispensable in the development of children.

From the aforementioned literature reviews, some general conclusions about the development of left-behind children’s SEC and its influencing mechanism have been drawn in the existing studies. Previous studies also had the following problems: first, studies on left-behind children focused more on a single dimension, such as parent–child attachment, peer relationship, and emotional development, but few of them used the SEC scale in the investigation and analysis. Second, some previous studies failed to take sample selection bias into account. The difference between left-behind children and non-left-behind children was not only reflected in the left-behind status but also reflected in other factors such as family capital. Some children may also be in a weak position in the development of the SEC even if their parents do not go out for work. If simple linear regression was directly used to compare the difference in SEC between left-behind children and non-left-behind children, the results may be biased. Third, existing studies have only considered it from the perspective of the family and lacked taking teacher support as a moderator variable to explore the moderating effect of teacher support on the development of children’s SEC. Therefore, based on the existing literature and the purpose of this study, the following assumptions were made in this study:

*H1*: Left-behind experience had a significant negative impact on children's SEC.

*H1a*: Teacher support could effectively mitigate the negative impact of left-behind children's SEC development.

*H2*: Family social, economic, and cultural capital positively affected left-behind children's SEC significantly.

**Figure 1:**
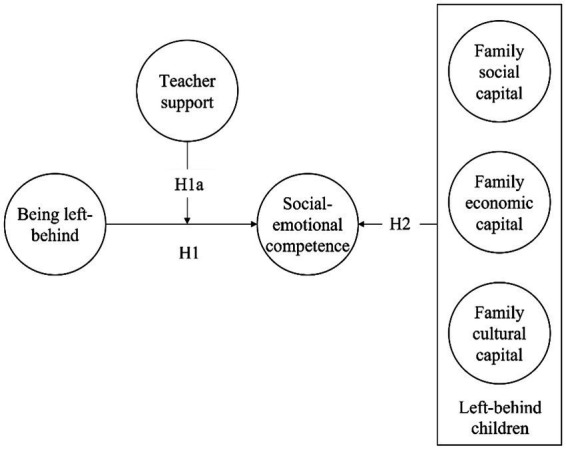
Conceptual framework of left-behind children SEC.

Drawing from the earlier assumptions, a conceptual model is plotted in Figure 1.

## 3. Research methods

### 3.1. Data sources

In this study, Hunan and Shandong provinces were randomly selected as the sources of the study group for this article among the provinces in China because they have a large number of migrant workers. As large labor-exporting provinces, Hunan and Shandong have a relatively large sample of left-behind children, and the samples were typical. A total of 578 valid questionnaires were obtained after eliminating invalid questionnaires such as missing values, abnormal values, conflicts between left-behind status and left-behind time, and samples of one or both parents who had passed away.

### 3.2. Instrument

#### 3.2.1. Dependent variable: Social–emotional competence

This study is adapted from the Delaware SEC scale (Mantz et al., 2018), which had 12 items and adopts a 4-point scoring method, on a scale of 1–4, indicating “Not at all like me,” “Not very like me,” “A little like me,” and “Very like me.” “I blame others when I get into trouble” was a reverse scoring, the other items were all positive scoring, and the higher the score the children get, the higher the children’s SEC. The questionnaire had been used in previous studies on rural children in China, with good reliability and validity. Cronbach’s alpha of this study was 0.769. The results of confirmatory factor analysis showed that all the indexes fit well, as shown: χ^2^/df = 1.499, RMSEA = 0.029, IFI = 0.983, TLI = 0.974, and CFI = 0.983.

#### 3.2.2. Independent variable: being left behind

Left-behind children refer to minors with rural household registration under the age of 16 years whose parents were out for work, or one of them was out for work and the other was incapable of guardianship and the left-behind children cannot live with their parents normally (Fan et al., 2010). Referring to this definition, this article defined the rural primary school students whose parents worked outside or were left at home as the left-behind children and assigned a value of 1. If both parents were at home or one of them was away from home and the other had guardianship ability, this study defined such rural primary school students as non-left-behind children, with a value of 0.

#### 3.2.3. Moderator variable: Teacher support

This study selected the teacher support questionnaire compiled by van de Pol et al. (2022) based on the growth experience and needs of left-behind children. There are teachers who will take an active interest in your emotional and academic state. “Teachers will listen to your thoughts and feelings,” “Teachers will talk to you about why things happen when you do something wrong and tell you the right ideas and practices,” “Teachers will actively take care of your school situation and offer guidance,” “Teachers will get help when you ask for help when you have learning problems,” “Teachers will encourage you when you encounter difficulties or setbacks,” and “Teachers will praise you when you do well,” using a 5-point scoring system, from 1 to 5, which meant “Strongly disagree,” “Disagree,” “Indeterminate,” “Strongly agree,” and “Agree.” The statistical correlation test showed that Cronbach’s alpha of this study was 0.761. The results of confirmatory factor analysis showed that **χ**^2^/df = 1.378, RMSEA = 0.026, IFI = 0.998, TLI = 0.995, and CFI = 0.998, and all the indexes fit well.

#### 3.2.4. Control variables

Based on the existing research, we took gender, grade, parents’ education, parents’ occupation type, and family economic condition as control variables. The occupations of parents were divided into two categories: elite occupations, which included managers of government agencies, institutions, and companies, as well as professional and technical personnel such as scientists, engineers, doctors, teachers, and information technology practitioners, and the rest of the occupations were classified as non-elite occupations. The family economic situation adopted five points, 1–5, which indicate “very rich,” “relatively rich,” “general,” “relatively poor,” and “very poor.”

### 3.3. Study design

When estimating the effect of left-behind experience on children’s SEC, the ideal method was to randomly assign the subjects to left-behind families or ordinary families in an experimental framework; thus, any difference in the outcome of children’s development can be attributed to the left-behind experience, or we could use a quasi-experimental study to test the difference in the results. In the absence of experiments or quasi-experiments, if covariates could be included in all the causes of left-behind children, the true impact of left-behind children could be estimated through statistical control of the children’s family background, but we could not capture all the factors that led to the consequences of being left behind. The propensity score matching (PSM) method alleviated this dilemma. We created a counterfactual group that was similar in observable characteristics to the experimental group to simulate randomization (Austin and Fine, 2019). The reason was that (a) it could assist in causal inference and correct for potential selection bias in the study, (b) the sample could meet the basic requirements for causal inference, and (c) the treatment effects obtained were close to the laboratory design.

The PSM model regressed the binary variables (left-behind children or not) and fitted a propensity value (PS value) for each sample, which represented the probability that a given sample was left-behind children, and the left-behind children and non-left-behind children were matched according to their propensity values. Its calculation formula is shown in (1), where X is the matching variable, i is the sample, and Z is left-behind children or the opposite (1 is left-behind children and 0 is non-left-behind children).


(1) $$PS\left(X_{i}\right)=P_{r}\left(Z_{i}=1|X_{i}\right)$$

In theory, there was no significant difference between left-behind children and non-left-behind children after the data balance. The only difference was whether they have left-behind experience. Therefore, the average treatment effect on the treated (ATT) could be calculated. Its calculation formula is shown in (2), where n1 represents the sample size of left-behind children, i and j represent indicators of the sample order of left-behind children and non-left-behind children, respectively, and i and j represent the weights of repeated matched control group samples.


(2) $$ATT=1/n^{1}\underset{i}{\sum }\left[\left(y_{i}|Z_{i}=1\right)\right]-\underset{i}{\sum }\omega_{i,j}\left(y_{i}|Z_{i}=0\right)$$

## 4. The results of the study

### 4.1. Descriptive statistical results

Descriptive statistics (Table 1) showed that there were 146 left-behind children (25.26%), among which boys accounted for 57%. There were 432 non–left-behind children (74.74%), among which boys accounted for 51%. In the father elite occupation variables, left-behind children accounted for 13% and non-left-behind children accounted for 16%. Mother was the elite occupation of equal proportion between left-behind children and non-left-behind children’s groups. The results showed that both left-behind children and non-left-behind children had good scores in SEC, while left-behind children had low scores in their father’s elite occupation and teacher’s support.

**Table 1:** Descriptive statistics of main variables.

Variable name	Full samples		Left-behind		Non-left-behind	
	*M*	SD	*M*	SD	*M*	SD
SEC	3.007	0.470	2.918	0.496	3.037	0.435
Boys^(c)^	0.530	—	0.570	—	0.510	—
Grade	1.880	0.632	1.950	0.481	1.860	0.675
Father’s elite career^(c)^	0.150	—	0.130	—	0.160	—
Mother’s elite career^(c)^	0.130	—	0.130	—	0.130	—
Father’s education	2.400	1.269	2.410	1.790	2.400	0.994
Mother’s education	2.410	1.011	2.410	0.940	2.410	1.034
Income situation	2.910	0.522	2.870	0.590	2.920	0.497
Stay on time	1.850	1.727	3.120	1.206	—	—
Teacher support	4.220	1.014	4.120	1.032	4.250	1.007
Sample size	578		146		432	

^(c)^The markers were all category variables, sex (female = 0, male = 1), occupation type (non-elite = 0, elite = 1), and the mean term indicates the proportion of this characteristic variable in the sample.

### 4.2. Benchmark regression result

As shown in Table 2, when the sample selection bias was not considered, the influence coefficient of left-behind on children’s SEC tended to decrease as the variables were continuously incorporated into the model. However, being left behind had a significant negative predictive effect on SEC, indicating that the left-behind children had a greater impact on the development of SEC. In addition, the ordinary least square (OLS) regression results also showed that different genders have significant differences in children’s SEC. Specifically, the SEC of left-behind boys was lower than that of girls. At the same time, the hindrance effect of mothers’ elite occupation on children’s SEC changed from significance to insignificance. To ensure the robustness of the regression results, the propensity score matching (PSM) method was used to further test the influence of being left behind on the development of children’s SEC, verifying the robustness and reliability of the results.

**Table 2:** Ordinary least square (OLS) regression results of the effect of being left behind on children’s SEC.

Variable	SEC								
Left-behind	−2.62***	−2.44**	−2.44**	−2.44**	−2.33**	−2.34**	−2.28**	−2.29**	−1.84*
	(0.047)	(0.047)	(0.047)	(0.047)	(0.047)	(0.047)	(0.047)	(0.047)	(0.050)
Boys		−2.57**	−2.58***	−2.60***	−2.65***	−2.66***	−2.63***	−2.63***	−2.64***
		(0.038)	(0.038)	(0.038)	(0.038)	(0.038)	(0.038)	(0.038)	(0.038)
Grade			−0.59	−0.61	−0.53	−0.47	−0.55	−0.54	−0.57
			(0.027)	(0.027)	(0.027)	(0.028)	(0.027)	(0.028)	(0.028)
Father’s elite career				0.57	1.05	0.89	0.81	0.75	0.70
				(0.054)	(0.055)	(0.056)	(0.056)	(0.056)	(0.056)
Mother’s elite career					−2.00**	−1.98**	−1.82*	−1.81	−1.76
					(0.066)	(0.066)	(0.066)	(0.066)	(0.066)
Father’s highest education						0.83	1.22	1.19	1.19
						(0.021)	(0.026)	(0.025)	(0.026)
Mother’s highest education							−1.00	−1.04	−1.04
							(0.026)	(0.027)	(0.027)
Income situation								−0.44	−0.55
								(0.040)	(0.040)
Stay on time									−0.92
									(0.012)
Region fixed effects	Control	Control	Control	Control	Control	Control	Control	Control	Control
_cons	116.83***	101.18***	48.63***	48.50***	49.20***	36.93***	36.65***	20.92***	20.80***
	(0.026)	(0.030)	(0.064)	(0.064)	(0.063)	(0.083)	(0.085)	(0.151)	(0.154)
*N*	576	576	576	576	576	576	576	576	576
*R* ^2^	0.014	0.025	0.025	0.026	0.035	0.036	0.039	0.039	0.040

(1) In parentheses were standard errors; (2) *, **, and *** were significant at the levels of 10, 5, and 1%, respectively.

### 4.3. PSM robustness test

#### 4.3.1. Common support hypothesis testing

**Figure 2:**
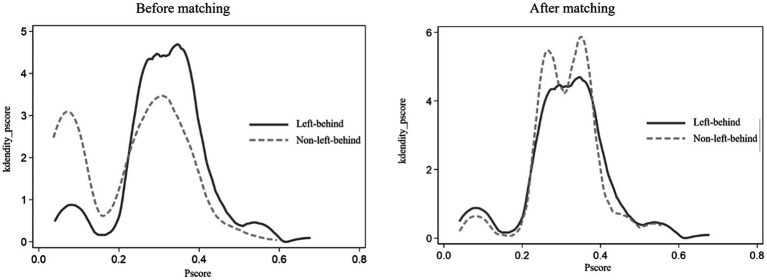
Matches the k-density *p*-scores.

The co-support hypothesis test and the balance test were performed to (a) balance the distribution of variables between the treated and the untreated and (b) ensure the rationality and validity of the PSM score results. Figure 2 was a kernel density (*k*-density) plot of the propensity score (*p*-score) before and after PSM matching. The propensity score density distribution of the treated group and the untreated group showed a more fitting trend after PSM matching. Most of the propensity scores of the two groups were in common support, indicating that the quality of matching was high. The difference between groups in observable eigenvalues was eliminated, which satisfied the common support hypothesis.

#### 4.3.2. The balance test

The results of the balance test in Table 3 showed that the pseudo-*R*^2^ after matching was only 0.007, the LR chi-square decreased from 56.99 to 2.62, the MeanBias decreased from 16.8 to 4.6 and the MedBias decreased from 11.4 to 4.2 (*p* = 0.977). The results showed that the self-selection problem resulted in a significant reduction of estimation bias in the matched sample, and the matching effect and estimation result was good.

**Table 3:** Matched the results of the pre and post-equilibrium joint tests.

Sample	Pseudo *R*^2^	LR chi^2^	*P* > chi^2^	MeanBias	MedBias
Raw	0.087	56.99	0.000	16.8	11.4
Matched	0.007	2.62	0.977	4.6	4.2

#### 4.3.3. Average treatment effect estimation

The nearest neighbor matching method was adopted to estimate the average treatment effect. The results showed that left-behind children had a significant negative impact on SEC. To test the reliability of PSM estimation, the results of radius matching, kernel regression matching, and local linear regression matching was further reported in Table 4. Left behind could reduce the scores of children’s SEC by 1.88, 2.46, 2.29, and 2.26, respectively. It could be seen that the results of all kinds of matching methods were larger than the estimated coefficients before matching, which indicated that sample selection bias may underestimate the effect of left-behind on children’s SEC.

**Table 4:** Processing effect of being left behind on children’s SEC.

Matching method	SEC		
	ATT	ATU	ATE
Nearest neighbor matching (*n* = 1)	−1.88*	−2.11**	−2.35**
	(0.066)	(0.056)	(0.051)
Radius matching (*r* = 0.01)	−2.46**	−2.46**	−2.46**
	(0.046)	(0.046)	(0.046)
Kernel regression matching	−2.29**	−1.84*	−2.07**
	(0.049)	(0.044)	(0.043)
Local linear regression matching	−2.26***	−1.54	−1.85*
	(0.050)	(0.045)	(0.043)

*, ** and *** indicated significant at the 10%, 5% and 1% levels respectively.

### 4.4. Heterogeneity analysis

#### 4.4.1. The influence of being left behind on the SEC of children of different genders

To further distinguish the gender differences in the impact of left-behind children on SEC, this study redivided the sample by gender. Table 5 shows that the effect of being left behind on children’s SEC was especially significant for boys.

**Table 5:** Heterogeneity analysis of the SEC development of left-behind children of different genders (ATT).

©	SEC	
	Boys	Girls
OLS	−1.90*	−0.83
	(0.066)	(0.073)
Nearest neighbor matching (*n* = 1)	−1.63*	−1.85*
	(0.092)	(0.093)
Radius matching (*r* = 0.01)	−2.22**	−1.12
	(0.065)	(0.072)
Kernel regression matching	−1.76*	−1.19
	(0.073)	(0.069)
Local linear regression matching	−1.85*	−1.10
	(0.071)	(0.075)

Heterogeneity analysis mainly reports ATT values. The standard error in parentheses is based on the standard error of 500 independent samples.

#### 4.4.2. The processing effect of left-behind duration on children’s SEC

Taking 1 year of being left behind as the limit reference, this study assigned a value of 0 for less than 1 year as the untreated group and a value of 1 for 1 year or more as the treated group. Through this analysis, we could further understand whether the increase in parents’ time out would be more detrimental to the development of left-behind children’s SEC. As shown in Table 6, children with more than 1 year of left-behind experience have a weaker SEC.

**Table 6:** Processing effect of left-behind duration on children’s SEC (ATT).

Matching method	SEC
OLS	−2.67**
	(0.089)
Nearest neighbor matching	−2.76***
	(0.131)
Radius matching	−2.86***
	(0.089)
Kernel regression matching	−2.76***
	(0.103)
Local linear regression matching	−2.56***
	(0.104)

** and *** indicated significant at the 5% and 1% levels respectively.

#### 4.4.3. The processing effect of family capital on left-behind children’s SEC

Children left-behind reflected the lack of social, economic, and cultural capital of migrant families. The development of children’s SEC was affected by the family capital. Therefore, it was out of practical significance to further explore the impact of family capital on children’s SEC. In this study, the family’s social, cultural, and economic capital were defined as the parents’ highest occupation type (non-elite occupation type = 0 and elite occupation type = 1), the parents’ highest education level (junior high school and below = 0 and senior high school and above = 1), and the family’s economic condition (very poor, relatively poor = 0, very rich, relatively rich, and general = 1).

According to Table 7, the scores of the left-behind children’s SEC have internal differences. The family cultural capital had a significant influence on the left-behind children’s SEC. On the contrary, the influence of parents’ occupation type and family economic condition on the left-behind children’s SEC was not significant.

**Table 7:** Processing effect of family background on left-behind children’s SEC (ATT).

Matching method	SEC		
	Occupation	Economy	Culture
OLS	−0.97	−0.89	2.53**
	(0.098)	(0.090)	(0.082)
Nearest neighbor matching	−1.15	−0.65	1.97**
	(0.124)	(0.122)	(0.097)
Radius matching	−0.39	−0.39	2.33**
	(0.108)	(0.094)	(0.083)
Kernel regression matching	−0.66	−0.53	2.16**
	(0.113)	(0.110)	(0.090)
Local linear regression matching	−0.53	−0.34	2.15**
	(0.112)	(0.108)	(0.089)

*, ** and *** indicated significant at the 10%, 5% and 1% levels respectively.

### 4.5. Adjustment effect test

Data were processed in SPSS, and the results are shown in Table 8. The interaction coefficient between teacher support and whether to stay was significant (*p* < 0.05), indicating that teacher support had a significant negative moderating effect on left-behind children.

**Table 8:** The moderating effect of teacher support on the SEC development of left-behind children.

Predictive variables	Model 1	Model 2	Model 3
	*β*	*β*	*β*
Constant	3.118***	3.091***	3.090***
	(0.068)	(0.064)	(0.064)
Gender	−0.115***	−0.104***	−0.110***
	(0.038)	(0.035)	(0.028)
Elite career for mothers	−0.094**	−0.076**	−0.075**
	(0.057)	(0.054)	(0.053)
Whether be left-behind		−0.091**	−0.094**
		(0.041)	(0.040)
Teacher support		0.338***	0.392***
		(0.020)	(0.021)
Whether be left-behind*teacher support			−0.093**
			(0.037)
*R* ^2^	0.156	0.385	0.392
Adjust *R*^2^	0.014	0.136	0.141
Δ*R*^2^	0.024**	0.124***	0.006**
Δ*F*	2.362***	41.430***	3.877**

Only significant control variables were shown in the model. ** and *** indicated significant at the 5% and 1% levels respectively.

## 5. Conclusion and discussion

Based on the survey data of senior primary school students in Hunan and Shandong provinces, this article constructed a PSM model. Nearest neighbor matching (*n* = 1), radius matching (*R* = 0.01), kernel regression matching, and local linear regression matching were used to analyze the influence effect and internal heterogeneity of left-behind children’s SEC, to explore the moderating effect of teacher support between left-behind and SEC.

The findings were as follows: first, being left behind can significantly negatively affect children’s SEC development, which was consistent with the findings of Wang et al. (2017). The results were still valid after a variety of matching methods, which verified hypothesis 1. Second, family cultural capital had a significant impact on children’s SEC, but family social capital and family economic capital have no significant impact. As Murray-Harvey and Slee (2007) suggested, students’ SEC was largely influenced by their families. However, the results of this study differed from those of Karabekiroglu et al. (2013), which showed that household economic capital, such as household income, was an important predictor of social–emotional problems. At the same time, several previous studies have shown (Mac Dermott et al., 2010; Appleton et al., 2012; He et al., 2019) that family social capital, such as the type of parental occupation, significantly affected children’s SEC. The possible reason was that previous studies have focused more on pre-schoolers. According to ecosystem theory (Bronfenbrenner, 1979), the development of microsystems of most pre-schoolers were limited to their family. Therefore, it was greatly influenced by the family income and the parents’ occupation. As the toddler grows into a school-going child, the scope of his activities continued to expand. As schools, teachers, and peers were integrated into this microsystem, the impact of the family gradually weakened. Therefore, for the students in the upper grades of primary school, the impact of family economic capital on SEC could be weakened. Finally, through the analysis of the moderating effect of teacher support, it was found that there was a significant correlation between the development of children’s SEC and teacher support. Furthermore, teacher support played a moderating role in the development of SEC of left-behind children, which verified hypothesis 3. This was consistent with the results of Alzahrani et al. (2019). In addition to the earlier hypothesis being verified, we also found that the influence of being left behind on children’s SEC was especially prominent in boys. The longer the boys are left behind in rural were as the worse the development of children’s SEC would be.

The earlier conclusions have the following implications. First, this study found that non-left-behind children’s SEC was significantly higher than left-behind children. There was a positive correlation between the time the children were left in rural areas and harm to the development of children’s SEC could not be independent of parental support. Parents play an irreplaceable role in children’s SEC development. Although some researchers have argued that with the advancement of society and communication technology, the negative effects of parent-child separation on left behind children can be compensated to some extent through video calls and voice calls. For example, the higher the frequency of parent-child communication, the less negative effects children face (Tang et al., 2021).

However, the results of this study showed that left-behind children still have much lower social and emotional competence than non-left-behind children in SEC. The possible reason was that the communication between parents who go out to work and left-behind children was fundamentally different from that of companionship at home. Previous studies have shown that family upbringing can significantly influence the development of children’s SEC (Chen et al., 2018). For parents working with lower income, reasonable parenting could benefit their future generations by narrowing the gap with the children of high-income families (Shepherd et al., 2021). This suggested that working parents need to pay more attention to raising their children rather than entrusting their children to the elderly or relatives. It was suggested that parents should adopt an equal and warm family education concept, which could build a bridge of “mutual trust” between parents and children through emotional warmth, encouragement, and support, to create a harmonious and safe atmosphere.

Second, we find that family cultural capital had a significant positive effect on left-behind children’s SEC, which was in line with Pierre Bourdieu’s theory of cultural capital. Cultural capital, as a kind of invisible capital, was considered to be a factor that can be passed on and implemented in the development of children. Moreover, cultural capital should cover all capital associated with culture and cultural activities rather than be defined simply by the single attribute of parents’ educational background. Therefore, we should attach importance to the family’s cultural capital construction to actively promote the cultural level of parents. Specific measures can be considered from the following aspects. The government and walks of life should accelerate the construction of high-quality educational infrastructure in rural schools, such as setting up village-run libraries and computer rooms and creating online resource learning spaces. To make up for the lack of family cultural capital, the scope of social service projects could be further expanded. Finally, the combination of government functions and social forces could accumulate long-term cultural capital for rural left-behind children.

For rural primary schools, the curriculum for the development of children’s SEC could be set up. Collaborative for Academic, Social, and Emotional Learning (CASEL) had developed relatively mature curriculums, including experience curriculum, pedagogical content knowledge curriculum, and hidden curriculum focusing on the creation of a school atmosphere (Xu et al., 2016). According to CASEL’s findings, students who took social–emotional classes showed significant improvements in academic performance, negative behavior, interpersonal relationships, and negative emotions. Children were ultimately guided to form positive moral and social perceptions (Ciotto and Gagnon, 2018; Mahoney et al., 2021). In addition, primary and secondary schools should conduct exchanges and cooperation with professionals in education and psychology at universities, such as inviting experts to offer courses in education and psychology. Meanwhile, some measures could be made for creating a novel, long-term cultural capital, such as carrying out monitoring and evaluation of the development of children’s SEC and establishing personal growth files for long-term tracking.

**Figure 3:**
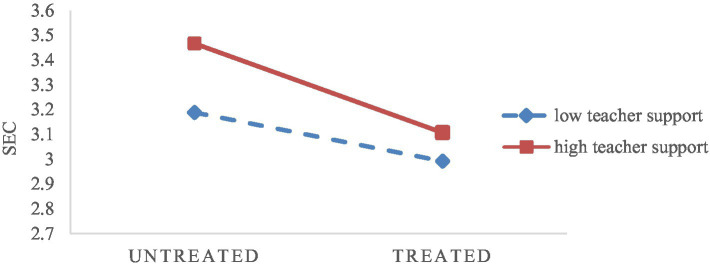
Moderating effect of teacher support on the relationship between left-behind and non-left-behind children’s SEC.

Third, for left-behind children whose parents were unable to take care of them, teacher support was an important factor affecting children’s SEC, which was aligned with Humphries et al. (2018). As shown in Figure 3, compared with low teacher support, both left-behind children and non-left-behind children had better scores in the high teacher support system. According to the field theory (Bourdieu, 2005), the development of an individual was influenced by many factors such as physical environment and behaviors. Obtaining high teacher support in schools helped students who had been separated from their parents for a long time to acquire new social capital, which could buffer the negative impact of being left behind on the development of children’s SEC. For a long time, the social concept of “academic performance first” was deeply rooted in people’s thoughts. Teachers pay more attention to children’s academic performance, and the lack of due attention to children’s SEC development, some left-behind children, fall into a vicious circle of “poor academic performance-low teacher support-weak SEC.” It was suggested that teachers should actively pay attention to and find out the problems left-behind children face in their growing up. Students’ social–emotional states need to be observed in time to meet students emotional and communication needs. At the same time, teachers should consciously give more support to students, rather than using coercive and command education methods, and promote the positive development of children’s SEC. However, since our society was focusing on promotion rates at present, teachers’ performance appraisals were geared toward children’s grades and promotion rates. This made teachers more likely to follow evaluation tests and unconsciously or forcefully ignore the development of social–emotional abilities of left-behind children. To encourage teachers to actively focus on social–emotional ability development among left-behind children under external objective conditions, it was suggested that non-cognitive ability development indicators, such as students’ social–emotional ability, be included in their appraisals and evaluations.

## 6. Research findings, limitations, and prospects

This study explored the influence mechanism of left-behind children’s SEC from four aspects: left-behind and non-left-behind, gender, family cultural capital, and teacher support. The results showed that (1) left-behind children had significant negative effects on SEC; (2) there were significant differences in the development of SEC among children of different genders and the duration of being left behind; (3) family cultural capital had significant positive effects on the SEC of left-behind children; and (4) teacher support had significant moderating effects on the influence path of “left-behind-SEC.”

The results of this study enriched the research on the development of children’s SEC and provided effective theoretical guidance for future efforts to promote the development of children’s SEC. However, there were some limitations. First, the development of children’s SEC in this study used a self-report questionnaire, which meant that there may be information bias in the data. Second, the study only collected data on children in some rural areas; thus, the sample coverage could be further expanded. Due to the difference in economic development between the east and the west of China, the regional sources of samples could be increased. Third, this study only analyzed the moderating effect of teacher support, and the mediating variables affecting children’s social-affective ability development deserve further investigation. In the future, more mediating variables can be included, and a chain mediating effect can be formed to further analyze the influencing mechanism of children’s SEC.

## Data availability statement

The raw data supporting the conclusions of this article will be made available by the authors, without undue reservation.

## Ethics statement

The studies involving human participants were reviewed and approved by Hangzhou Normal University. Written informed consent to participate in this study was provided by the participants’ legal guardian/next of kin.

## Author contributions

XM: conceptualization, data curation, formal analysis, investigation, methodology, manuscript writing, and supervision. CJ: conceptualization, investigation, supervision, manuscript writing, funding acquisition, and project administration. GS: formal analysis, methodology, and manuscript writing. YZ and XX: investigation, manuscript writing, and review editing. All authors contributed to the article and approved the submitted version.

## Funding

This article was supported by a general project of the National Social Science Fund of China (The year 2021, Project Number: BHA210133).

## Conflict of interest

The authors declare that the research was conducted in the absence of any commercial or financial relationships that could be construed as a potential conflict of interest.

## Publisher’s note

All claims expressed in this article are solely those of the authors and do not necessarily represent those of their affiliated organizations, or those of the publisher, the editors and the reviewers. Any product that may be evaluated in this article, or claim that may be made by its manufacturer, is not guaranteed or endorsed by the publisher.
